# Molecular signatures between citrus and *Candidatus* Liberibacter asiaticus

**DOI:** 10.1371/journal.ppat.1010071

**Published:** 2021-12-09

**Authors:** Bin Hu, Muhammad Junaid Rao, Xiuxin Deng, Sheo Shankar Pandey, Connor Hendrich, Fang Ding, Nian Wang, Qiang Xu

**Affiliations:** 1 Key Laboratory of Horticultural Plant Biology (Ministry of Education), Key Laboratory of Biology and Genetic Improvement of Horticultural Crops (Ministry of Agriculture), Huazhong Agricultural University, Wuhan, Hubei, China; 2 Citrus Research and Education Center, Department of Microbiology and Cell Science, Institute of Food and Agricultural Sciences, University of Florida, Lake Alfred, Florida, United States of America; 3 Hubei Key Laboratory of Plant Pathology, Huazhong Agricultural University, Wuhan, Hubei, China; University of Basel, SWITZERLAND

## Abstract

Citrus Huanglongbing **(**HLB), also known as citrus greening, is one of the most devastating citrus diseases worldwide. *Candidatus* Liberibacter asiaticus (*C*Las) is the most prevalent strain associated with HLB, which is yet to be cultured in vitro. None of the commercial citrus cultivars are resistant to HLB. The pathosystem of *Ca*. Liberibacter is complex and remains a mystery. In this review, we focus on the recent progress in genomic research on the pathogen, the interaction of host and *C*Las, and the influence of *C*Las infection on the transcripts, proteins, and metabolism of the host. We have also focused on the identification of candidate genes for *C*Las pathogenicity or the improvements of HLB tolerance in citrus. In the end, we propose potentially promising areas for mechanistic studies of *C*Las pathogenicity, defense regulators, and genetic improvement for HLB tolerance/resistance in the future.

## Introduction

Citrus is one of the most important fruit crops cultivated in at least 114 countries around the world [1]. Citrus is usually prone to suffer from various diseases due to the lack of diversity caused by asexual reproduction and propagation [2]. Huanglongbing (HLB) is the most devastating citrus disease worldwide. Most commercial citrus cultivars are susceptible to HLB [3] with varying degrees of symptoms [4–6]. HLB has been reported in most citrus producing areas, such as Africa (e.g., Ethiopia and Reunion Island), the Americas (e.g., United States, Mexico, Brazil, and Cuba), Oceania [7], and Asia (e.g., China, India, and Pakistan) [8], except for the Mediterranean region and Australia [7]. HLB causes billion-dollar annual losses to the citrus industry [3,9–12]. In China, HLB was first discovered in Chaoshan, Guangdong Province [8]. In the USA, HLB was first reported in Florida in 2005 [13]. By 2018, most mature citrus trees have been infected with the HLB pathogen in Florida, causing approximately 75% reduction in citrus production compared with that in 2005 [14]. In Brazil, approximately 1 million citrus trees were removed within several months since HLB was first reported in São Paulo in 2004 [15]. China, Brazil, the US, and many other citrus-growing countries have invested large amounts of research funding and organized cooperative research among scientists in the fields of citrus, pathology, and entomology to prevent and control this epidemic disease. The combined efforts from global scientists and other stakeholders have made people aware of the severity of the disease. The scientific community is exploring wild germplasms or citrus relative species to improve the HLB resistance and tolerance of citrus cultivars.

Structural and functional genomics studies advance our understanding of the HLB pathosystem. Here, we reviewed the recent studies of *C*Las pathogen and the host plant. We have briefly described the genomes of *Ca*. Liberibacter and explained how the bacterium induces pathogenicity, multiplication, and influence on host genetic activities. We also deliberated the host genetic, transcript, and proteomic changes in response to the bacterium. Moreover, we have proposed some potential steps to minimize the *C*Las pathogenicity and the promising areas for host genetic improvements and future opportunities to improve citrus tolerance for HLB disease.

## 1. Symptoms and disease cycle of HLB

**Fig 1:**
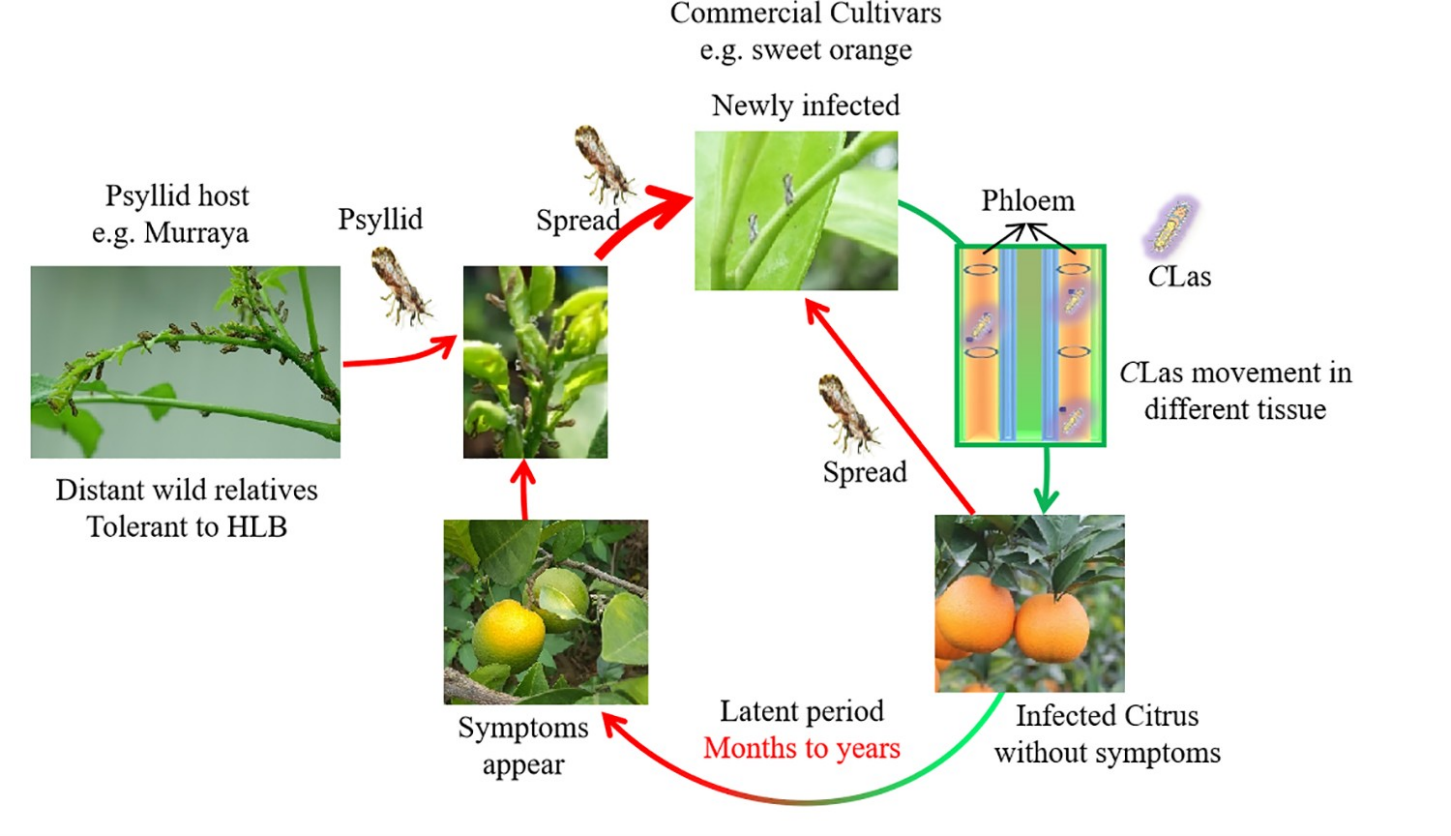
Disease cycle of citrus HLB. *C*Las has a wide range of hosts, and almost all citrus varieties and relatives can be infected. *C*Las is transmitted by ACPs. All citrus species can be the host of ACP [27,28]. After latent period (months), the citrus trees show mild to severe HLB symptoms [20]. ACP, Asian citrus psyllid; HLB, Huanglongbing.

HLB is mainly associated with *C*Las, *Candidatus* Liberibacter africanus (*C*Laf), and *Candidatus* Liberibacter americanus (*C*Lam) [3], among which *C*Las is the most predominant pathogen in most citrus-producing regions and has certain degrees of tolerance to heat, while *C*Laf and *C*Lam are heat sensitive [16]. *C*Laf is mainly distributed in Africa, and *C*Lam was only reported in Brazil [17]. The insect, psyllid (*Diaphorina citri*), is the most influential vector to transmit the disease (Fig 1).

Due to the long latency period of HLB, the *Ca*. Liberibacter–infected citrus trees do not show visible symptoms at the initial stage of infection. Moreover, the latency period is usually several months long [3,11,18–21] but varies depending on citrus variety, tree age, health status, and the environmental factors [9], which gives rise to asymptomatic infections to cause the widespread of the disease. Citrus plants infected by *Ca*. Liberibacter usually display symptoms of stunted growth, root decay, thinner canopy, yellow shoot, blotchy mottle leaves, upright and small leaves, early flowering, and an overall tree decline [3,22–26]. Infected fruits are often small and lopsided with uneven coloration [12]. Some mandarin varieties produce “red nose fruit” or “red shoulder fruit” due to the orange-red color in the fruit shoulder while cyan and dull color in other parts of the fruit. The disease cycle of HLB is represented in Fig 1.

## 2. Transient culture of *Ca*. Liberibacter

*Ca*. Liberibacter associated with HLB have not been cultured in artificial media [3,11]. The uncultivable characteristic of the pathogen in vitro heavily hampers mechanistic studies of pathogenicity. Hence, tremendous efforts have been made in the culture of *Ca*. Liberibacter. *C*Las and actinobacteria (*Propionibacterium acnes*) was reported to coculture in artificial media [29]. In vitro, *C*Las and *P*. *acnes* can survive multiple passages together. However, *C*Las is unable to grow when cultured independently [29]. This suggests that *P*. *acnes* might facilitate *C*Las growth. *C*Las can live for several weeks in vitro with the addition of commercial grapefruit juice to the medium; and it is noteworthy that some microbes (including *C*Las and other bacteria) can be found in grapefruit seeds [30]. In addition, *C*Las was reported to be able to grow for several months inside the biofilms formed by other bacteria [30]. *C*Las from Hamlin sweet orange extract was cultured and maintained over 2 years along with microbiota inside a membrane biofilm reactor supplemented with specific nutrients composition [31]. The 2 species *C*Las and *C*Lam were reported to grow on Liber A medium [32]. Recently, the *C*Las strain Ishi-1 with phages [33] was cocultured with phloem-associated microbiota in vitro. The growth of Ishi-1 was determined based on the identification of *C*Las population and increase of *C*Las DNA amounts; however, there has been no direct evidence to support the phenomenon of Ishi-1 growth [34]. *C*Lso and *C*Las were able to be maintained in vivo in hairy root explants for 28 and 120 days, respectively [35]. Similarly, the leaf discs with supplemented glucose show an increase in *C*Las titer [36]. Thus, these studies suggested that other endogenous microbes may facilitate *C*Las growth and colonization in citrus.

*Liberibacter crescens* (Lcr), a Liberibacter species newly identified from defoliated Babaco mountain papaya (*L*. *crescens* strain BT-1), can be cultured, but its pathogenic activity has not been reported so far [37]. A comparison of the Lcr genome with that of other Liberibacter species showed that Lcr possesses more genes encoding thiamine and essential amino acids [37], which might explain why Lcr is culturable whereas other Liberibacter species are not. Genomic comparisons between *C*Las and Lcr revealed important information about the missed genes in *C*Las genome that prohibit the growth of *C*Las in artificial media, which may not be easily solved by adjustments of the compositions of media [38]. All pathogenic and unculturable Liberibacter species have no functional glyoxalase pathway, but this pathway is present in Lcr, which prevents both prokaryotes and eukaryotes from proteome glycation and methylglyoxal-induced carbonyl stress [39]. When infecting either plants or psyllids, due to the lack of the *gloA* gene in the genome, *C*Las can circumvent a toxic buildup of cellular methylglyoxal pool by preventing sugar uptake and glycolysis [39]. Therefore, addition of specific methylglyoxal-binding compounds to the culture medium [40] or transferring *gloA* gene from Lcr to *C*Las has been suggested to be a possible way to culture *C*Las in axenic media [38]. In addition, homologous genes of *LpxXL* and *AcpXL*, encoding a very long chain fatty acid (VLCFA)-modified lipid A, are present in Lcr (*WP_015273388*.*1* and *WP_015273393*.*1*) but absent in all pathogenic and unculturable Liberibacter species [37]. Mutation of the *Lcr lpxL* gene was lethal [41], suggesting that the VLCFA-modified lipid A is essential for the axenic growth of pathogenic Liberibacter species.

The mutualistic relationship between *C*Las and other bacteria suggests that *C*Las may obtain essential nutrients and/or some active substances from other bacteria for its own growth [38]. The differences in genome between *C*Las and Lcr likely determine why Lcr is culturable in artificial media while *C*Las is not. It remains to be determined whether it is possible to make *C*Las culturable or to make Lcr pathogenic to investigate the HLB pathosystem.

## 3. Genomes of *Candidatus* Liberibacter species

*Ca*. Liberibacter is a phloem-colonizing gram-negative bacterium [3,16] belonging to the Rhizobiaceae family of *α*-Proteobacteria [16]. The genetic diversity analysis of *C*Las on genes, such as the *β*-operon gene loci, *omp* gene [42,43], and 16S rRNA [44,45] indicated the rapid speciation occurred for Liberibacter species. Genome sequencing has been accomplished for 6 Liberibacter species, including *C*Las, *C*Laf, *C*Lam, *Ca*. Liberibacter solanacearum (*C*Lso), Lcr, and *Ca*. Liberibacter europaeus (*C*Leu). These genomes range from 1.15 to 1.52 Mb in size with low GC contents from 31.1% to 36.6%. The phylogenetic analysis of 36 Liberibacter species and 8 *Rhizobiale* species suggested that they first evolved from a common ancestor into nonpathogenic Lcr, followed by the evolution to pathogenic *Ca*. Liberibacter [46]. The genome sequence of *C*Las suggests that it is an early-branching member of the *Rhizobiaceae* family [47]. A comparative genomic analysis has revealed that the regulatory network of *C*Las is rather simple, with only 11 transcription factors in the entire transcriptome [47]. Some genomic features of *Ca*. Liberibacter species are summarized in Table 1 and S1 Table.

**Table 1:** The genomic features of sequenced *Ca*. Liberibacter species.

Species	Strain	BioProject	Area	Level	Size (Mb)	GC (%)	Gene	Pseudo-gene	Reference
*C*Las	A4 gxpsy psy62 JXGC Ishi-1 AHCA1 JRPAMB1 CoFLP TaiYZ2	PRJNA239529 PRJNA158395 PRJNA29835 PRJNA376787 PRJDB1752 PRJNA470611 PRJNA544530 PRJNA638026 PRJNA552755	Guangdong, China Guangxi, China Florida, USA California, USA Ishigaki (Island), Japan California, USA Florida, USA La Guajira, Colombia Thailand	Complete Complete Complete Complete Complete Chromosome Complete Complete Complete	1.23025 1.26824 1.22733 1.22516 1.19085 1.23375 1.23717 1.23164 1.23062	36.4 36.6 36.5 36.4 36.3 36.6 36.4 36.5 36.4	1125 1159 1120 1113 1076 1107 1113 1104 1104	28 28 37 24 23 39 21 27 23	[53] [54] [47] [55] [56] [57] [58] [59] [46]
*C*Lso	CLso-ZC1 LsoNZ1 FIN114 HenneA RSTM FIN111 ISR100 R1	PRJNA39273 PRJNA243548 PRJNA312061 PRJNA259360 PRJNA298929 PRJNA312579 PRJNA427973 PRJNA251993	Texas, USA Northland, New Zealand south-west Finland Texas, USA California, USA south-west Finland Israel California, USA	Complete Contig Contig Contig Contig Contig Contig Contig	1.25828 1.31242 1.24512 1.21136 1.28679 1.2024 1.30369 1.20426	35.2 35.3 35.2 34.9 35.1 34.9 35 35.3	1145 1203 1132 1102 1181 1091 1210 1111	68 53 59 158 63 45 65 112	[60] [61] [51] [61] [62] [51] [63] [64]
*C*Lam	PW_SP Sao Paulo	PRJNA185961 PRJNA181147	São Paulo State, Brazil São Paulo State, Brazil	Contig Complete	1.1952 1.17607	31.1 31.1	1007 1028	26 26	[65] [66]
*C*Laf	PTSAPSY	PRJNA185151	South Africa	Complete	1.19	34.5	981	54	[67]
*C*Leu	ASNZ1	PRJNA243548	Canterbury, New Zealand	Contig	1.33	33.5	1179	35	[68]
Lcr	BT-0 BT-1	PRJNA269727 PRJNA171392	Puerto Rico Puerto Rico	Complete Complete	1.52212 1.50466	35.4 35.4	1376 1359	17 65	[37] [37]

The genomes of *C*Lbr and *C*Lso haplotype U are not available when this review is written.

*C*Lso comprises 6 haplotypes, including haplotype A-E [48,49] and U [50]. Haplotype A and B are capable of causing diseases in solanaceous plants such as the Zebra chip disease of potato, and haplotype C, D, and E are associated with diseases of apiaceous plants [51]. The *C*Lso haplotype U was found in the psyllid *Trioza urticae* and its host plant *Urtica dioica*, and called “U” after *Urtica* [50]. *Ca*. L. brunswickensis (*C*Lbr) was first identified in the Australian eggplant psyllid (*Acizzia solanicola*), and its genomic sequence remains not publicly available [52]. The publication of more genome sequences of Liberibacter species will promote more robust analyses. These genome sequences will enable the genomic comparison among various Liberibacter species, which can facilitate a better understanding of the lifestyle of *Ca*. Liberibacter species and the interactions between *C*Las and citrus plants.

## 4. Genes affecting *C*Las growth and pathogenicity

The understanding of pathogenicity of HLB bacteria has advanced much with the availability of *Ca*. Liberibacter genomes and rapid development of comparative genomics and functional genomics. We summarized advances in nutritional metabolism, prophage, and secretion systems below.

### 4.1. Metabolic genes

Metabolic model reconstruction analysis of 6 *C*Las strains indicated that most of the common essential genes are involved in purine and pyrimidine metabolism, pantothenate and CoA biosynthesis, fatty acid metabolism, and gluconeogenesis [69]. *C*Las has the ability to metabolize sugars such as glucose, fructose, and xylulose but not mannose, galactose, rhamnose, or cellulose [47]. The concentrations of fructose and glucose are very low in the phloem sap [70,71]; therefore, consumption of fructose by *C*Las during infection may initiate a shift in the host metabolite distribution [47]. Metabolomic studies have suggested broad changes in sugar concentrations in *C*Las-infected tissues including depletions of glucose and fructose in the leaves and symptomatic fruits of multiple citrus varieties (at 8 months post inoculation) [72–74]. However, in some of these studies, sugar levels changed significantly at different time points during infection [72,73]. Dissecting changes directly caused by *C*Las sugar consumption versus effects of the overall disruption of host biology would require further study. *C*Las disrupts the host cellular metabolic functions by importing multiple metabolites from host for its growth and development that leads to severe disease symptoms.

The reduced genome size of *C*Las indicates that the pathogen heavily depends on the host nutrition [10]. The presence of large number of transporter proteins in *C*Las might play a critical role in providing *C*Las with necessary nutrients and cause a metabolic imbalance in citrus [10]. *C*Las contains 14 ABC transporter-related proteins that help the bacterium import metabolites (amino acid and phosphates) and enzyme cofactors (choline, thiamine, iron, manganese, and zinc) from host [75] and resist organic solvent, lipid-like drugs and heavy metal; maintain the composition of the outer membrane; and secrete virulence factors [10]. The phosphatidylcholine (PC) (synthesized by Phospholipid N-methyltransferase and phosphatidylcholine) is associated with the fluidity, permeability, and potential of bacterial membranes [76], and the proteins for the 2 pathways of PC biosynthesis can be found in *Lcr* (*WP_015272535*.*1* and *WP_015272978*.*1*, respectively), while *C*Las lacks the ability to synthesize PC. However, *C*Las encodes a predicted ABC transporter system for choline (*CLIBASIA_01125*) and a phosphatidylcholine synthase (*CLIBASIA_02325*) [75], suggesting that it is capable of utilizing extracellular choline.

### 4.2. Secretion-related genes

*C*Las lacks type III and type IV secretion systems and some related enzymes involved in extracellular living [47]. The genes related to cell motility such as those encoding flagellin and type IV pili account for as much as 4.5% of the *C*Las genome [47]. All the genes associated with type I secretion system required for both toxin effector secretion and multidrug efflux are present in the *C*Las genome [47]. The Sec pathway is involved in the translocation of proteins from the cytoplasm into the periplasmic space [77,78]. Interestingly, a complete Sec pathway and type I section system are encoded by *C*Las [47]. The type I secretion pathways have 2 primary functions. The first one is defensive, protecting the bacterium against toxic environmental chemicals, involving multidrug efflux, antibiotics produced by other bacteria, and guarding against the phytoalexins produced by hosts [47]. Multidrug efflux has been demonstrated as an important mechanism for bacterial survival in members of the following genera *Xanthomonas*, *Erwinia*, *Bradyrhizobium*, *Agrobacterium*, and *Rhizobium* [79]. The second one is offensive, allowing the secretion of a number of offensive effectors and degradative enzymes, some of which are involved in plant or animal pathogenicity and others are antibiotics [47]. Offensive effectors and enzymes known to be secreted via the type I system include a relatively large number of protein toxins, including RTX hemolysins and bacteriocins and a limited number of hydrolases (esterases, proteases, glucanases, phosphatases, and nucleases) [80,81]. The type I secretion systems in gram-negative bacteria are typically composed of 3 protein components, *TolC*, which traverses both the outer membrane and periplasm, and 2 others, membrane fusion protein, MFP, and ATP-binding cassette, ABC, which are localized in the inner membrane [81,82]. Generally, the phytopathogenic bacteria possess multiple copies of *TolC*; however, *C*Las genome possesses only 1 copy of *TolC* [47]. Similarly, the *Xylella* genome also possesses 1 copy of *TolC* [79]. Knockout of *TolC* gene makes *Xylella* totally nonpathogenic and highly sensitive to phytoalexins [79], raising the possibility of a gene-engineered or chemical approach to target the single *TolC* gene of *C*Las.

### 4.3. Prophage

Three complete and 1 remnant (Type 4) prophages have been found in *C*Las genomes, including Type 1 (SC1), Type 2 (SC2) [83,84], Type 3 (P-JXGC-3) [55], and Type 4 [46]. SC1 encodes a putative holin and endolysin [83] that is localized outside the predicted prophage region, showing the potential for lytic and lysogenic change. SC2 encodes putative adhesin and peroxidase genes, which may be involved in lysogenic conversion [84]. The variants of SC1 and SC2, named P-PA19-1 and P-PA19-2, respectively, were found in *C*Las strain PA19 from Pakistan [85]. The results of expression (*SC1_gp100*, *SC1_gp025*, *SC1_gp110*, and *SC1_gp095*) and transformation with the fusion of the holin promoter region and a *uidA* reporter in *Lcr* have suggested that the activation of *C*Las prophage may reduce the host plant range and culturability of *C*Las [83]. The lytic burst of *C*Las in living phloem cells might trigger the death of the phloem cells [10], which seems to explain the phenomenon that no *C*Las is observed in infected citrus leaf midribs during the advanced stages of HLB [4]. A study has revealed that small Wolbachia protein may play a role as the repressor of *C*Las prophages, but the lytic cycle was still found in citrus psyllid [86]. Type 3 prophage of *C*Las has been identified to be incapable of reproduction via lytic cycle [55]. Type 3 prophage of *C*Las has 50% unique genes compared with SC1 and SC2 prophages and carries a restriction–modification system, which was speculated to play a role against Type 1 prophage/phage invasion [55]. Type 4 prophages have been found in *C*Las, *C*Lam, *C*Laf, and *C*Leu, and they differ in Las isolates with the presence or absence of other phages [33,46]. *C*Lam genome has 2 prophages, SP1 and SP2, whereas *C*Laf genome has only one [46,66]. *C*Leu and *C*Lso both harbor 2 prophages [46]. In addition, 2 homologous genes *lasA*_*I*_ and *lasA*_*II*_ (previously named as *hyv*_*I*_ and *hyv*_*II*_) were discovered in *C*Las Psy62 genome, which might trigger high levels of genetic variability in plant immune response [87]. The prophage region in *C*Las genome psy62 encodes a putative protein (123 amino acids) named as *CLasP235*, and overexpression of *CLasP235* in Carrizo (*Citrus sinensis* × *Poncirus trifoliata*) was reported to result in HLB-like symptoms and chlorosis. In addition, grapefruit and lemon chlorotic leaves infected by *C*Las also showed high expression of the *CLasP235* gene [88].

## 5. The interaction between *C*Las and host plant

### 5.1. PTI and ETI

Pathogen-associated molecular patterns (PAMPs) are found in or associated with disease-causing microorganisms, which mainly include bacterial DNA, lipoteichoic acids in the cell wall, and lipopolysaccharides [89–91]. The plant innate immunity system is core for the interaction between microbes and plant hosts [90]. This system constitutes at least 2 layers. PAMP-triggered immunity (PTI) is one of the first layer of the immunity that can recognize PAMPs and activate defense signaling or gene expression, which reinforces the physical barriers against the pathogen attack, such as callose reinforcing the cell wall at sites of infection, and production of reactive oxygen species [90–92]. The second layer is effector-triggered immunity (ETI). ETI commonly deploys disease resistance (R) proteins for effective counteraction against effectors [90]. PTI is mediated by pattern recognition receptors (PRRs) that recognize PAMPs, whereas ETI is mediated by resistance (R) proteins that recognize pathogen effectors [90,91]. Additionally, in citrus HLB disease, the role of ETI and PTI immunity systems is not well defined. The flagellin-encoding gene *flaA* (*CLIBASIA_02090*) [93], which was identified as a PAMP in *C*Las, may play a crucial role in triggering host plant resistance to the infection of *C*Las [89]. Type IV pili can induce inflammatory responses in animal hosts and cell death in nonhost plant upon infection by pathogenic bacteria, respectively [94,95]. *Ca*. Liberibacter spp. also encode the complete set of genes required for Tad type IV pili synthesis and assembly [96]. The in planta expression of type IV pili and their interaction with host plants need further investigation. Few PAMPs in *C*Las have been reported, and the mechanism of *C*Las pathogenesis remains elusive.

### 5.2. Virulence factors of *C*Las

Investigation of the effectors and finding or identification of some binding proteins in citrus may provide an alternative and more sustainable way to block the invasion of *C*Las. Pathogen recognition by the plant immune system leads to defense responses that are often accompanied by a form of regulated cell death known as the hypersensitive response (HR) [97]. HR can be uncoupled from local defense responses at the site of infection, and its role in immunity may activate systemic responses in distal parts of the organism [97]. A large-scale screen of the virulence factors of *C*Las using *Tobacco mosaic virus* (TMV) and *Nicotiana benthamiana* reveals that *CLIBASIA_05150* and *CLIBASIA_04065C* (C-terminal of *CLIBASIA_04065*) could trigger cell death, and symptoms of stunting are observed in the plants expressing *CLIBASIA_00470* and *CLIBASIA_04025* [98]. Callose deposition is an important plant multifaceted defense mechanism (controlled by distinct signaling pathways) that acts to reinforce plant cell wall at the site of pathogen attack [99]. The mature protein of *CLIBASIA_00460* (m460) is localized in multiple cellular compartments including nucleus at 25°C, but nuclear accumulation of m460 is dramatically decreased at 32°C [100]. NLS-m460, containing the SV40 nuclear localization sequence (NLS) at the N-terminus to promote nuclear import of m460, triggers chlorosis and necrosis in the local leaves and severe necrosis in the systemic leaves in *N*. *benthamiana* [100].

The overexpression of *CLIBASIA_03875* [101] and *CLIBASIA_04405* [102] mature protein via a *Potato virus* X (PVX)-based expression vector in *N*. *benthamiana* suppressed programmed cell death (PCD) and H_2_O_2_ accumulation triggered by the proapoptotic mouse protein BAX and the *Phytophthora infestans* elicitin INF1 and contributed to the symptoms of dwarfing, leaf deformation, and mosaics. Simultaneously, *CLIBASIA_03875* was the first PCD suppressor identified from *C*Las [101]. Approximately 27 nonclassically secreted proteins (ncSecPs) were identified from *C*Las genome, using the SecretomeP program coupled with an *Escherichia coli* alkaline phosphatase assay [103]. Among which, 10 of these were dramatically more highly expressed in citrus than in psyllid and particularly suppressed HR-based cell death and H_2_O_2_ accumulation in *N*. *benthamiana* [103].

### 5.3. Interaction of *C*Las and citrus

*C*Las5315mp (mature protein), which is encoded by *CLIBASIA_05315*, is localized in the chloroplast and induces cell death in *N*. *benthamiana* and callose deposition in plant cells [104]. *C*LasΔ5315 with the removal of the chloroplast transit peptide from the *C*Las5315mp induces excessive starch accumulation in *N*. *benthamiana* [105]. Additionally, *CLIBASIA_05315* might be the most promising gene that can be used as a marker for early detection of HLB, as it is expressed in asymptomatic tissues [106]. In a separate study, the effector Sec-delivered effector 1 (SDE1) encoded by *CLIBASIA_05315* directly interacts with papain-like cysteine proteases (PLCPs) and inhibits protease activity [107]. Meanwhile, SDE1 interacts with DDX3 and down-regulates the expression of DDX3 in HLB-affected yellowing and mottled leaves of citrus, causing HLB typical chlorosis symptoms [108], which, however, was not observed by Clark and colleagues [107]. Severe yellowing and senescence signatures were observed in the mature leaves of SDE1-expressing *Arabidopsis thaliana* lines [109]. Moreover, SDE1-expressing Duncan grapefruit exhibited hypersusceptibility to *C*Las [109]. The expression of PR genes (*PR1*, *PR3*, and *PR5*) and PTI marker genes (*FRK1*, *GST1*, and *WRKY22*) was significantly down-regulated after XccA^w^ inoculation in the SDE15-transgenic compared to that in the nontransgenic Duncan grapefruit [110]. SDE15 interacts with citrus protein CsACD2 (accelerated cell death 2), which encodes a chlorophyll catabolite reductase that represses PCD in plants [111]. The virulence factor SDE15 might be a broad-spectrum suppressor of plant immunity, which suppresses the HR induced by *Xanthomonas citri* subsp. citri (*Xcc*) in the transgenic Duncan grapefruit of SDE15. SDE15 also suppresses the HR triggered by the AvrBsT (*Xanthomonas vesicatoria* effector protein) in *N*. *benthamiana* [110].

## 6. Host responses to *C*Las infection

Different responses are associated with different citrus germplasms when infected by *C*Las, including pathogen titers, the severity degree of symptoms, and the time of symptom appears [6]. Generally, citrus germplasm is considered as HLB tolerant when the pathogen is detectable but with low titers and the plant exhibits no or slight symptoms, which do not affect normal development. Susceptible species usually have high pathogen titers and typical disease symptoms [5,6]. The rapid advancement of genomic approaches facilitated the understanding of how *Ca*. Liberibacter infection affects transcriptome [112–120], proteome [121,122], and metabolome [123–125] in host plant.

### 6.1. Transcriptional change

Plants species possess a different set of genes that respond to a variety of abiotic and biotic stresses and provide local or systemic defense responses against pathogens [126]. These defense genes usually belong to transcriptional factors, pathogenesis-related (PR), protease inhibitors (PIs) gene families, and some are related to secondary metabolites to produce antimicrobial compounds [127–129]. The salicylic acid (SA) biosynthesis and induction of different defense responses (*WRKY*, *PR*, and *PI*) vary from species to species, and it also depends on the type and intensity of pathogen [130,131]. Interestingly, several transcriptomic studies on HLB-infected citrus revealed that citrus species express multiple genes that belong to *WRKY* (*WRKY33*, *WRKY40*, *WRKY41*, *WRKY46*, and *WRKY70*), *PR*, and secondary metabolic categories in response to *C*Las invasion [132–134]. Induced expression of *PR1* gene is a marker for SA-mediated defense signaling pathway [135]. Additionally, an increased level of jasmonic acid and SA was observed after *C*Las infection [136]. Analysis of biotic response-associated DEGs from asymptomatic and symptomatic stages of the relatively tolerant Mexican lime (*Citrus aurantifolia*) suggests the role of secondary metabolism, cell wall, signaling, transcription factors, and redox reactions in HLB tolerance [137]. The wide-range gene expression analyses of *C*. *sinensis*, *Citrus sunki*, *P*. *trifoliata*, and contrasting hybrids representing susceptible, tolerant, and resistant varieties against HLB suggest that the down-regulation of gibberellin synthesis, induction of cell wall strengthening, and enhanced expression of WRKY transcription factors are associated with tolerance against *C*Las infection [138,139]. The changes in gene expression related to photosynthesis, carbohydrate metabolism, glucose transportation, and starch synthesis/degradation are presumed to lead to starch accumulation after *C*Las infection [140–142]. In addition, those genes associated with cell defense and cell wall were also differentially expressed after *C*Las infection [23,120,143]. Some regulators on transcriptional or posttranscriptional levels were also targeted. *WRKY40*, *NAC* domain, and *MYB15* may play important roles in regulating carbohydrate metabolism and defense response in citrus–*C*Las interactions [132]. miR399 is induced by the infection of *C*Las [132], which responds to phosphorus starvation in other plant species [144]. The phosphorus content of *C*Las-infected citrus was 35% lower than that of the healthy control. Application of phosphorus oxyanion solutions to HLB-infected citrus was reported to alleviate the symptom severity and improve fruit production [144], indicating that HLB may result in phosphorus deficiency. Some HLB-responsive miRNAs, such as *csi-miR167*, are associated with potassium (K) transport. In addition, K-deficient citrus plants are more prone to *C*Las invasion than those plants with abundant K supply [132,144].

The developmental stages of the leaves of host plants are crucial for the *C*Las pathosystem [21,145–148]. Young leaves at advanced developmental stages display enhanced constitutive expression of immunity-related genes, which may provide additional tolerance to bacterial infections [149]. Host responses against *C*Las infections vary in different tissues of same plants. The virulence, stress response, and antimicrobial secondary metabolites-associated genes show enhanced expression in midrib tissues from the leaves compared to the fruit piths in HLB-positive *Citrus reticulata* Blanco “Shatangju” [150]. Similarly, the temporal host response may vary in citrus against *C*Las infestation. The signaling, defense, transcription factors, hormone, and photosynthesis pathways are differentially expressed even at day 1, and DEG bursts occur for genes related to secondary metabolites, defense, photosynthesis, and glycolytic and ATP biosynthetic pathways at 5-day post-ACP-mediated *C*Las inoculation [151].

### 6.2. Protein change

A wide range of alterations were observed at the proteomic level such as suppression of heat shock proteins and metabolism- and photosynthesis-related proteins, which may facilitate *C*Las invasion. Down-regulation of some key proteins such as photosynthesis- and metabolism-related proteins and photosystem II reaction PSB28 protein [152] may cause chlorosis and host environments favorable for HLB progression. Eventually, pathways involved in the photosystem I and II light reactions are suppressed throughout the *C*Las infection process [113]. Symptomatic fruit of Valencia sweet orange exhibit less accumulation of proteins involved in amino acid biosynthesis, glycolysis, and tricarboxylic acid (TCA) cycle [153]. Phloem proteome analysis of Washington navel orange, an HLB-susceptible sweet orange variety, shows decreased expression of proteins of plant metabolism and translation but enhanced expression of defense-related proteins, including proteases, PIs, and peroxidases [154]. Several proteins involved in photosynthesis are less accumulated and are proposed to be responsible for the reduction of Ca, Mg, Fe, Zn, Mn, and Cu contents in infected grapefruit leaves [155–157], which may explain the nutrient deficiency of HLB-infected trees. In contrast, the proteins involved in cell wall modification are more accumulated in HLB tolerant citrus species [151,152], such as expansin β-3.1, pectinesterase, CESA8, and expansin8 [152]. Mexican lime expressing β-defensin 2 and/or lysozyme showed lower bacterial titers and less severe HLB-like symptoms as well as increased photosynthesis compared with the control trees [158]. In the young leaves of citrus, CSLG2, UGE5, expansin4, RGP2, and glycoside hydrolase family 28 proteins are more accumulated by *C*Las. However, in tolerant citrus germplasms, thylakoid luminal 20 kDa protein, chlorophyll binding, oxygen-evolving complex-related, and 2 ferredoxin-related proteins are suppressed [152]. The proteins associated with the detoxification of oxidative stress (nitrilases and glutathione S-transferases) and cell wall and PR proteins are activated, demonstrating their potential to boost HLB tolerance in citrus [121,122].

### 6.3. Metabolite changes

A large number of primary and secondary metabolites are involved in maintaining the normal functions and immune response of plants [159]. *C*Las unbalance the primary metabolism of HLB susceptible varieties, and the host fails to activate its secondary defense system, whereas in HLB-tolerant varieties, the primary metabolism is balanced, which can coordinate with other defense pathways to respond to *C*Las invasion [160]. Generally, the tolerant varieties have high levels of flavonoids (such as flavonols and flavones) with strong antibacterial properties and amino acid precursors to defensive phenolic compounds (phenylalanine, tryptophan, and tyrosine), whereas susceptible varieties lack these compounds [124]. Probably, high concentrations of flavonoids [161,162] and volatile compounds in the tolerant varieties may contribute to their tolerance against *Ca*. Liberibacter [124]. Curiously, some fatty acids associated with defense were strongly depleted in infected sweet orange leaves [163]. Whether these fatty acids are depleted due to host defense responses or are offensively destroyed by *C*Las is unclear, as does the role of these fatty acids in HLB progression. Modification of the metabolic pathways for higher contents of antibacterial metabolites will be an alternative strategy to enhance HLB tolerance of the susceptible citrus germplasm.

## 7. Improvements for HLB tolerance

Different approaches have been investigated with the aim to improve citrus tolerance against HLB. A synthetic, high-throughput screening system is performed to identify compounds that inhibit activity of *C*Las transcription activators LdtR, RpoH, and VisNR [164]. Among 120,000 compounds screened from this system, 5 compounds are validated to have inhibitory effects on one or several of the *C*Las transcriptional activators [164]. A study also showed that naturally occurring flavonoids had inhibitory effects on YbeY activity in *C*Las [165]. The in silico and experimental analysis of genes overexpressed during *C*Las infection reveals that 8 enzymes including DTMP kinase, inorganic diphosphatase, coproporphyrinogen oxidase, protoporphyrinogen oxidase, phosphoglycerate mutase, dihydroorotic acid, ribonucleoside-diphosphate reductase (UDP) (glutaredoxin), and glutaredoxin reductase’s inhibition could reduce *C*Las pathogenicity, thus providing potential genetic targets in the *C*Las strains [69].

Given the importance of Sec translocon and its substrates, inhibition of the Sec secretion system by antimicrobial agents with suitable targets such as SecA can suppress the progression of HLB [166]. A recent study identified a stable antimicrobial peptide (SAMP) from *Microcitrus* that can inhibit *C*Las infection, which effectively reduced disease symptoms in HLB-positive trees but also induced innate immunity [167]. Appropriate application of antimicrobial agents against *C*Las may be an alternative approach to boost HLB tolerance by activating the expression of some proteins involved in radical ion detoxification [151].

**Fig 2:**
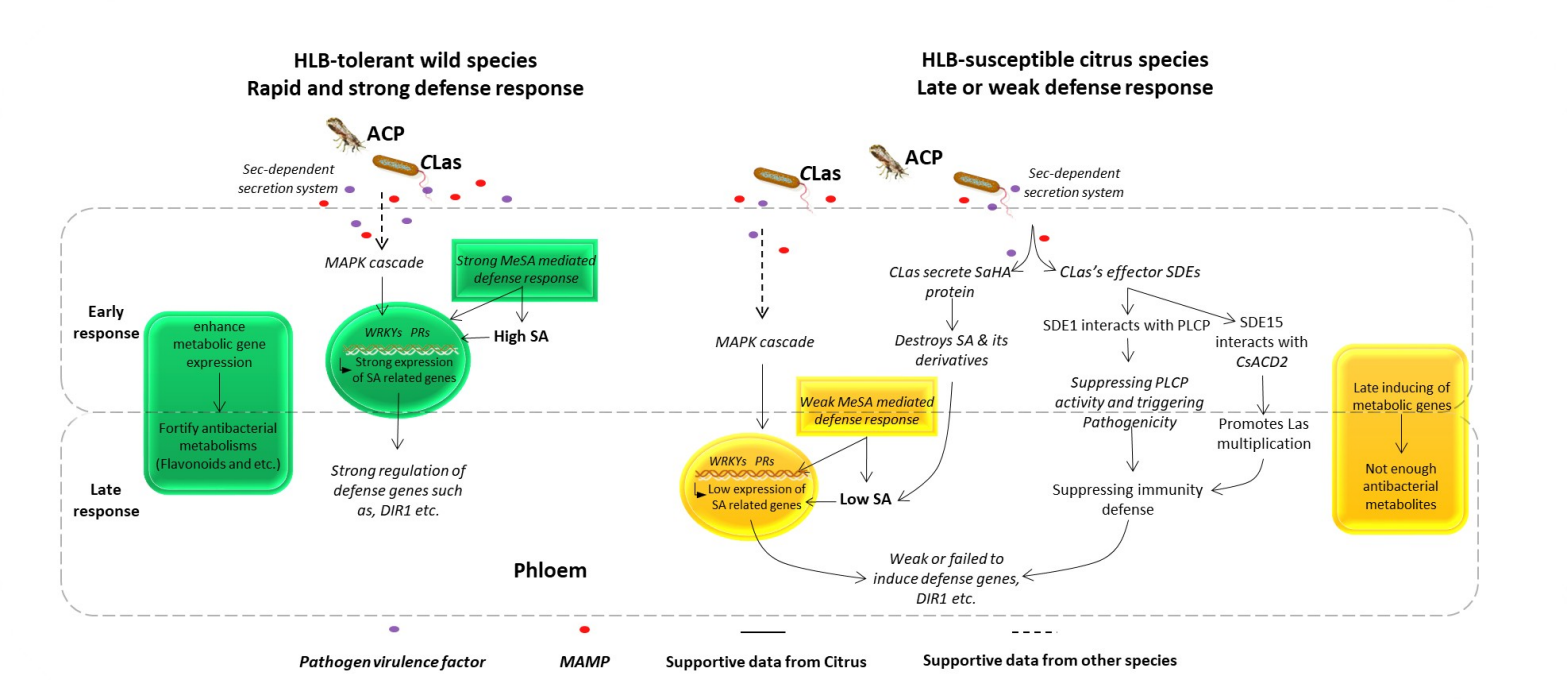
Sketch of the hypothetical pathway of HLB-tolerant and HLB-susceptible citrus species in response to *C*Las. In HLB-tolerant citrus species, first, *C*Las invasion causes cell signaling, which enhances the secondary metabolic genes [160] to biosynthesize antimicrobial compounds such as volatiles, fatty acids, amino acids, and some antibacterial compounds such as flavonol, flavone, and flavanone [124]; second, *C*Las may secrete PAMPs and pathogen virulence factors into the phloem to interfere with various targets [7] such as genes, proteins, and metabolites. In HLB-tolerant citrus, the MAPK [133] activates the downstream defense-related genes such as WRKY genes to trigger the PR reaction and strongly induce SA-mediated defense response [168], and the expression of *DIR1* genes, NPR4, SA-related genes will be induced to contribute to the high HLB tolerance [143]. In HLB-susceptible citrus species, first, *C*Las infection affects the photosynthesis and primary metabolism, decreases starch degradation enzymes, increases the expression of starch biosynthetic genes (such as *GBSS1* and *glgC*), and induces *PP2* gene, which triggers starch and callose accumulation and causes phloem plugging [118]. Disruption of primary metabolism causes delayed or reduced biosynthesis of secondary metabolites (antibacterial compounds such as flavonols), and susceptible citrus shows severe symptoms; second, the *C*Las secretes virulence factor proteins such as a functional enzyme SahA into citrus plant, destroying the host’s SA and its derivatives to suppress the host defense [116]. In addition, SDEs move into cells via the Sec-dependent secretion system; SDEs such as SDE1 interact with receptor protein PLCP and suppress its activity, which weakens the citrus plant defense response [107]. Moreover, the SDE15 interacts with citrus protein CsACD2 and suppresses the plant immunity and promotes *C*Las multiplication [110]. In this way, the *C*Las protein disrupts the normal metabolism and defense system of host cells by modifying the host cellular machinery to manipulate pathogenicity and to make the host environment favorable for *C*Las survival and progression. ACP, Asian citrus psyllid; HLB, Huanglongbing; MAPK, mitogen-activated protein kinase; PAMP, pathogen-associated molecular pattern; PLCP, papain-like cysteine protease; PR, pathogen-related; SA, salicylic acid; SahA, salicylate hydroxylase; SDE, Sec-delivered effector.

Genetic and genomic studies focused on HLB tolerance species also provided candidate genes for disease resistance improvement. *P*. *trifoliata* and its hybrids, US-942 (*C*. *reticulata* “Sunki” *× P*. *trifoliata*) [169] and US-897 (*C*. *reticulata* × *P*. *trifoliata*) [169,170], are identified as tolerance germplasm [6]. QTL analysis conducted on HLB-tolerant *Poncirus*, and its intergeneric F_1_ population with sweet orange resulted in 3 repeatable QTL clusters (linkage groups LG-t6, LG-t8, and LG-t9). Most of detected QTLs could explain 18% to 30% of phenotypic variance [171]. The comparative analysis of small RNA profiles and target gene expression between an HLB-tolerant citrus hybrid (*P*. *trifoliata* × *C*. *reticulata*) and a susceptible citrus variety identified a panel of candidate defense regulators for plant immune responses against HLB, such as the positive regulator BRCA1-associated protein and the negative regulator vascular-associated death protein [172]. *C*Las infection and *D*. *citri* infestation noticeably increase endogenous melatonin levels in citrus leaves and up-regulate the expression of its biosynthetic genes (*CsTDC*, *CsT5H*, *CsSNAT*, *CsASMT*, and *CsCOMT*) [173]. Importantly, melatonin supplementation enhances the endogenous contents of the stress-associated phytohormones (salicylates, auxins, *trans*-jasmonic acid, and abscisic acid) and the transcript levels of their biosynthetic genes and diminishes the *C*Las titer in the infected leaves, which suggests that melatonin might play an antibacterial role against *C*Las [173]. SA may play a role in citrus defense against *C*Las, as it is commonly found to be increased in infected tissue [112,113], and artificially increasing SA levels can increase tolerance to HLB [114,115]. Perhaps because of this important defensive role, *C*Las encodes a salicylic hydrolase (SahA) presumably to break down host SA [10,116]. Based on the summarization of this review, the responses of molecular pathways of HLB-tolerant and HLB-susceptible citrus species to *C*Las infection are proposed in Fig 2.

Genome editing such as CRISPR-Cas technologies can be used for genetic manipulation, which provides an unprecedented opportunity to improve HLB tolerance [7]. Additionally, multiple genes can be edited by means of multiplex CRISPR with one single insertion [125]. Therefore, editing and improvement of defense regulators, genes encoding antibacterial compounds, or genes essential for the interaction between *C*Las and host species may constitute good strategies to enhance HLB tolerance [124]. For example, the citrus PLCPs genes could be used for such purpose. PLCPs, which encode immune-related cysteine proteases, were reported to be targeted and inhibited by *C*Las. Hence, it is necessary to characterize citrus PLCPs gene function in citrus. Moreover, identification of HLB-susceptible genes may also be useful. To date, 9 genomes of citrus have been published and are publicly available (http://citrus.hzau.edu.cn/; https://phytozome-next.jgi.doe.gov/), which include varieties with different degrees of tolerance to HLB disease. Besides, public availability of HLB-tolerant and HLB-sensitive citrus germplasm will facilitate the mining of the susceptible genes to be edited with the latest genome engineering tools.

## 8. Future prospects

Currently, HLB management strategy involves psyllid control, removal of HLB-diseased trees, and replantation with HLB-free trees for citrus-producing regions with low HLB incidence (Region-wide comprehensive implementation of rouging infected trees, tree replacement, and insecticide applications successfully controls citrus HLB). The strategy has positive consequences in controlling of HLB spreading; however, breeding of HLB-resistant or highly tolerant cultivars is a fundamental way to solve this devastating disease. Researchers from all over the world have made tremendous efforts to study the HLB pathosystem, and great progress has been made in the exploration of HLB-tolerant genetic resources, the pathogenic mechanism of *C*Las, and the behavior of psyllid. In the next 5 to 10 years, gene mining, especially for the pathogenic effectors and host interaction genes, will become a hot spot of research, and further understanding of the resistant/tolerant and susceptible mechanism against HLB will facilitate the development of genetic techniques to improve HLB resistance. Breakthroughs may be expected to be made from the following aspects.

First, the pathogenicity of *C*Las, *C*Lam, and *C*Laf needs to be further dissected; the effector and their target proteins should be clearly pinpointed. Genome sequencing, comparison, and functional analysis will gain new knowledge on the pathogenicity and in vitro culture. Synthetic biology including different chimeric genomes will be innovative approaches to speed up the advance in this area.

Second, genes involved in the early interaction between *Ca*. Liberibacter and host should be clarified. Most of the current molecular data are from weeks or even months after infection. There has been rather limited evidence from the immediate effect (e.g., hours after infection or within 1 day) on citrus plants caused by *C*Las infection. Some progress has been made in early detection of the damage caused by HLB [106,174–177]. Identification of the early damage caused by HLB infection is pivotal for the elucidation of its pathogenic mechanism.

Third, the population of *Ca*. Liberibacter may comprise benign strains with potential biological controlling effects against virulent strains. Thus, a global and continuous monitoring of the *Ca*. Liberibacter population and their virulence will be of significant data in this area.

Fourth, it is promising to enhance tolerance in commercial citrus by using HLB-tolerant wild citrus by precising selection with molecular breeding. Citrus relatives (*Murraya paniculata* and *Atalantia buxifolia*) and wild citrus (*Citrus latipes* or *Poncirus trifoliata*) are highly tolerant to HLB. Multiomics methods can be utilized to analyze the molecular basis of HLB tolerance and then identify the key candidate genes for HLB tolerance.

Last, metabolic improvement and optimization may be an alternative way to increase HLB tolerance. Significant changes were observed in metabolic pathways after the infection of HLB bacteria. The key metabolites or regulators can be screened with metabolomics, and their antibacterial effects should be experimentally confirmed.

## Supporting information

S1 Table The information of unassembled genomes for the *C*Las. (DOCX)
