# Supplementary material for: Molecular signatures between citrus and Candidatus Liberibacter asiaticus
Source: PLoS Pathog. 2021 Dec 9;17(12):e1010071. doi: 10.1371/journal.ppat.1010071 (PMC8659345; doi:10.1371/journal.ppat.1010071)
Supplement: S1 Table — (DOCX) [file ppat.1010071.s001.docx]

S1 Table. The information of un-assembled genomes for the *C*Las.

|  |  |  |  |  |  |  |  |
| --- | --- | --- | --- | --- | --- | --- | --- |
| **Species** | **Strain** | **BioProject** | **Level** | **Size(Mb)** | **GC(%)** | **Gene** | **Pseudo-gene** |
| *C*Las | 9PA  A-SBCA19  CHUC  CRCFL16  DUR1TX1  DUR2TX1  FL17  GFR3TX3  HHCA  HHCA16  JXGZ-1  LBR19TX2  LBR23TX5  Mex8  MFL16  PA19  PA20  SGCA1  SGCA16  SGCA5  SGpsy  TX1712  TX2351  YCPsy  YNJS7C  YNXP-1  AHCA17 | PRJNA624158  PRJNA665711  PRJNA561387  PRJNA561388  PRJNA561394  PRJNA561393  PRJNA269509  PRJNA561380  PRJNA244958  PRJNA561386  PRJNA552376  PRJNA561391  PRJNA561392  PRJNA561396  PRJNA561389  PRJNA593634  PRJNA593355  PRJNA470611  PRJNA561390  PRJNA299501  PRJNA470611  PRJNA451209  PRJNA361117  PRJNA292590  PRJNA488522  PRJNA551602  PRJNA552562 | Contig  Contig  Contig  Contig  Contig  Contig  Contig  Contig  Contig  Contig  Contig  Contig  Contig  Contig  Contig  Contig  Contig  Contig  Contig  Contig  Contig  Contig  Contig  Contig  Contig  Contig  Scaffold | 1.23188  1.18688  1.20845  1.20828  1.20629  1.21232  1.22725  1.20932  1.15062  1.20705  1.21799  1.20275  1.20348  1.24313  1.19922  1.22416  1.22622  0.233414  1.20995  1.20138  0.769888  1.20333  1.252  1.23365  1.25899  1.20707  1.20862 | 36.7  36.3  36.4  36.5  36.4  36.3  36.5  36.4  36.5  36.3  36.4  36.3  36.4  36.4  36.5  36.4  36.4  36.3  36.4  36.4  36.3  36.4  36.5  36.5  36.6  36.3  36.4 | 1185  1109  1103  1158  1084  1146  1103  1094  1150  1102  1088  1074  1075  1136  1119  1096  1102  515  1090  1099  NA  NA  1166  1108  1135  1082  1096 | 102  67  28  83  32  96  30  39  228  46  22  25  25  51  66  27  24  7  34  49  NA  NA  93  27  17  24  32 |
|  |  |  |  |  |  |  |  |
